# Hydrodynamic instabilities provide a generic route to spontaneous biomimetic oscillations in chemomechanically active filaments

**DOI:** 10.1038/srep01964

**Published:** 2013-06-11

**Authors:** Abhrajit Laskar, Rajeev Singh, Somdeb Ghose, Gayathri Jayaraman, P. B. Sunil Kumar, R. Adhikari

**Affiliations:** 1 The Institute of Mathematical Sciences, CIT Campus, Chennai 600113, India; 2 Department of Physics, Indian Institute of Technology Madras, Chennai 600036, India

## Abstract

Non-equilibrium processes which convert chemical energy into mechanical motion enable the motility of organisms. Bundles of inextensible filaments driven by energy transduction of molecular motors form essential components of micron-scale motility engines like cilia and flagella. The mimicry of cilia-like motion in recent experiments on synthetic active filaments supports the idea that generic physical mechanisms may be sufficient to generate such motion. Here we show, theoretically, that the competition between the destabilising effect of hydrodynamic interactions induced by force-free and torque-free chemomechanically active flows, and the stabilising effect of nonlinear elasticity, provides a generic route to spontaneous oscillations in active filaments. These oscillations, reminiscent of prokaryotic and eukaryotic flagellar motion, are obtained without having to invoke structural complexity or biochemical regulation. This minimality implies that biomimetic oscillations, previously observed only in complex bundles of active filaments, can be replicated in simple chains of generic chemomechanically active beads.

Prokaryotic bacteria^1^ as well as eukaryotic sperm cells^2^,^3^ employ rhythmic flagellar beating for locomotion in viscous fluids. Bacterial flagella rotate rigidly in corkscrew fashion^4^,^5^, while spermatic flagella behave more like flexible oars^6^ with their beating mostly confined to a plane^7^,^8^,^9^. Oscillatory motility in clamped flagella can arise spontaneously and, with an unlimited supply of energy, can persist indefinitely without any external or internal regulatory pacemaker mechanism^3^,^10^. Autonomous motility as well as spontaneous beating due to hydrodynamic instabilities has been recently reproduced *in vitro*^11^,^12^, where a biomimetic active motor-microtubule assemblage has been shown to exhibit remarkable cilialike beating motion with hydrodynamic interactions (HI) playing a crucial role in synchronised oscillations^11^. Previous models^13^,^14^,^15^,^16^,^17^,^18^,^19^,^20^,^21^,^22^,^23^,^24^ analysing the mechanism behind flagellar beating have, in general, ignored the role of HI.

Here we study a minimal active filament model^25^ which, once clamped at one end, exhibits a variety of spontaneous beating phenomena in a three dimensional fluid. Our model filament consists of chemomechanically active beads (CABs) which convert chemical energy to mechanical work in viscous fluids. These CABs are connected through potentials that restricts extensibility and enforces semiflexibility and self-avoidance of the filament. The conversion of chemical energy to mechanical work within the fluid produces flows which do not add net linear or angular momentum to it and, thus, must be represented at low Reynolds numbers by force-free and torque-free singularities^8^,^26^,^27^,^28^,^29^,^30^. We model the activity of the beads by a stresslet singularity which produce a flow decaying as 1/*r*^2^. This stresslet contribution arises from chemomechanical activity, for instance the metachronal waves of ciliated organisms^11^, or from phoretic flows in synthetic catalytic nanorods^31^,^32^,^33^,^34^. For self-propelled particles, additional dipolar contributions generating flows decaying as 1/*r*^3^ are present, but are neglected here as they are subdominant to stresslet contributions. The equation of motion for the active filament^25^ incorporating the effects of nonlinear elastic deformations, active processes and HI is 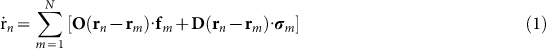
where **r***_n_* is the location of the *n*-th bead, **f***_n_* is the total elastic force on the *n*-th bead, and 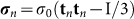 is stresslet tensor directed along the the local unit tangent **t***_n_*. Here *σ*_0_ > 0 sets the scale of (extensile) activity. The monopolar Oseen tensor **O** and the dipolar stresslet tensor **D** respectively propagate the elastic and active contributions to the flow (details of model in Supplementary Text). Noise, of both chemomechanical and thermal origin, can be added to these equations, but are not considered here. We impose clamped boundary conditions at one end and solve the equation of motion through direct summation of the hydrodynamic Green's functions. For a filament of length *L* and bending modulus *κ* the dynamics is characterised by the dimensionless activity number 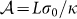 25.

## Results

### Spontaneous oscillations

We briefly recall the mechanism behind hydrodynamic instabilities in active filaments^25^. Extensile activity in a straight filament produces flows with dipolar symmetry that point tangentially outward at the filament ends and normally inward at the filament midpoint. A spontaneous transverse perturbation breaks flow symmetry about the filament midpoint resulting in a net flow in the direction of the perturbation. The destabilising effect of the hydrodynamic flow is countered by the stabilising effect of linear elasticity for activity numbers 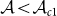 but leads to a linear instability for 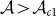. This instability produces filament deformations which are ultimately contained by the non-linear elasticity producing autonomously motile conformations^25^. Here, the additional constraint imposed by the clamp transforms the autonomously motile states into ones with spontaneous oscillations. We perform numerical simulations of the active filament model to show that the interplay of hydrodynamic instabilities, non-linear elasticity, and the constraint imposed by the clamp leads to spontaneously oscillating states.

**Figure 1:**
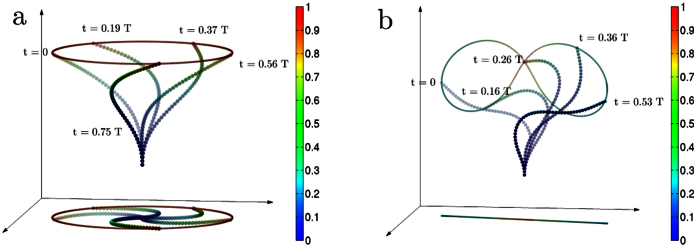
Biomimetic oscillations of the clamped filament plotted at different times over an oscillation period *T*. In (a) we see rigid aplanar corkscrew rotation for 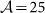 while in (b) we see flexible planar beating for 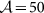. The colour of the beads as well as the trace of the tip correspond to individual instantaneous monomer speeds. The colourbars are normalised by the maximum speed.

**Figure 2:**
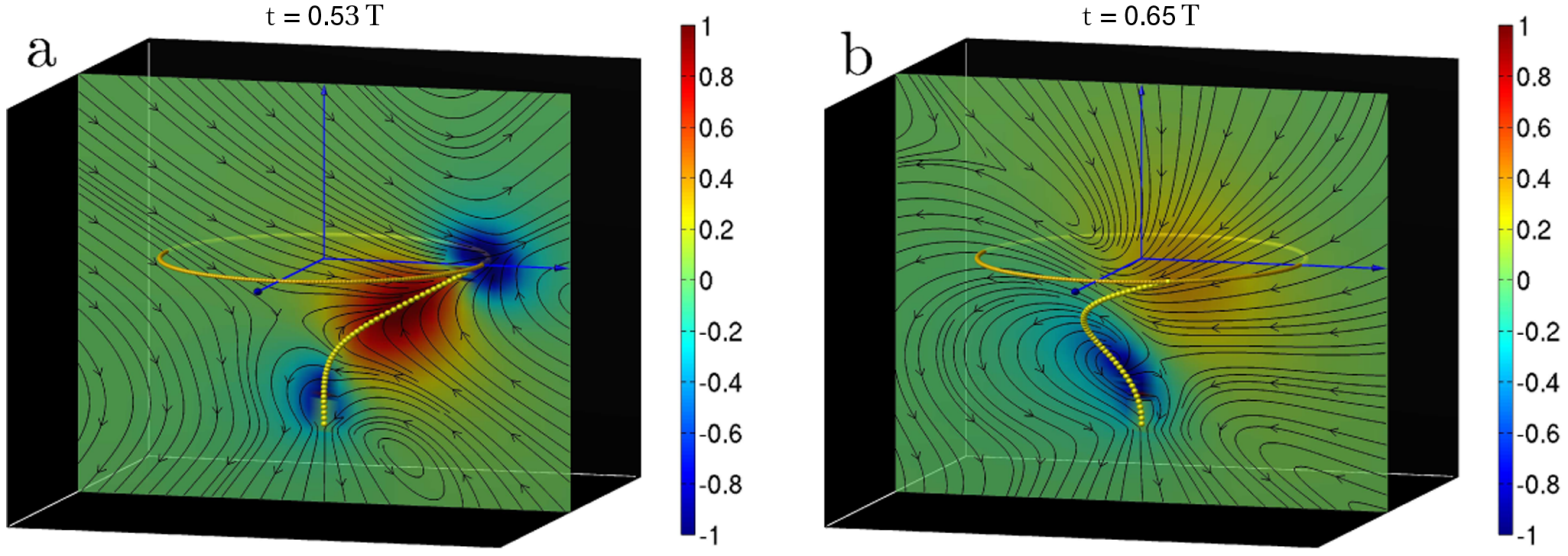
Flow fields of rigid aplanar corkscrew rotation at two different instants of the oscillation cycle. The colour indicates the signed magnitude of the velocity field perpendicular to the plane normalised by its maximum.

**Figure 3:**
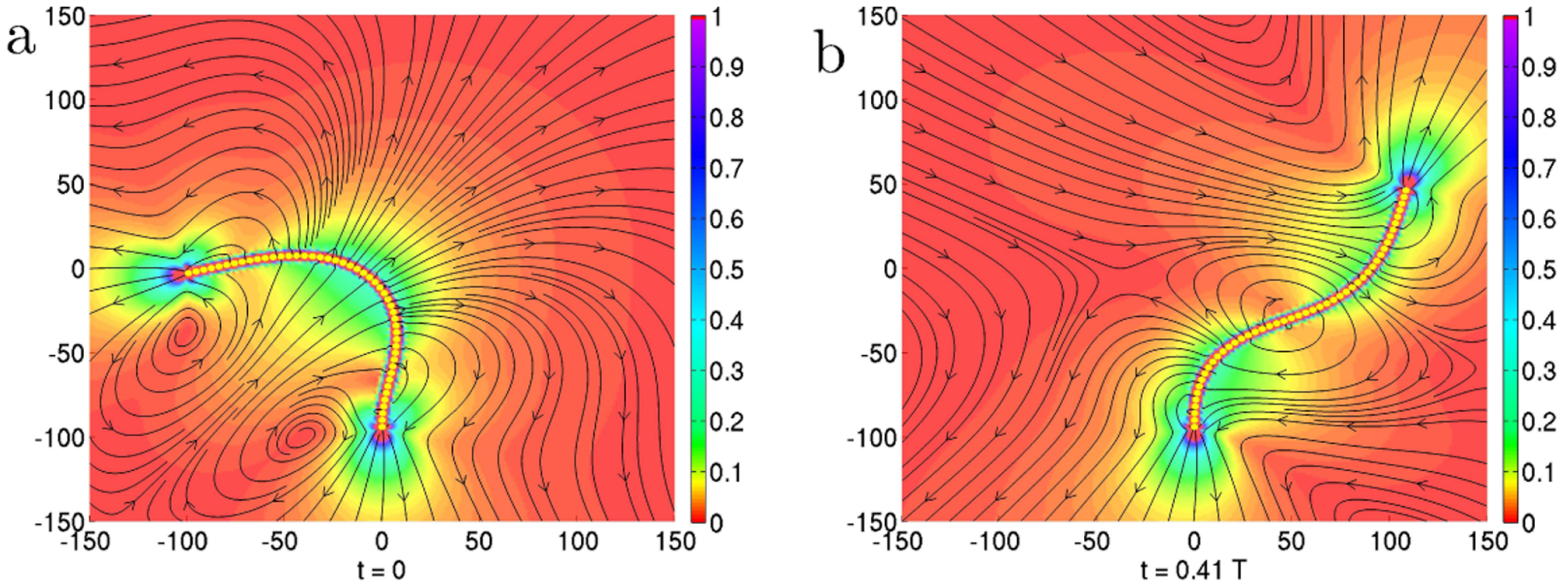
Flow fields of flexible planar beating at two instants of the oscillation cycle. The colour indicates the magnitude of the velocity in the plane normalised by its maximum.

**Figure 4:**
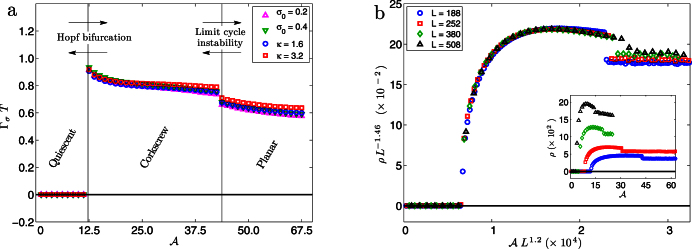
Variation of the scaled timeperiod Γ*_σ_T* of filament beating with 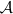 plotted for various values of *κ* and *σ*_0_ with *L* = 188, and (b, main panel) variation of the scaled amplitude *L*^–1.46^*ρ* with 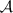 plotted for various lengths *L* with *κ* = 1.6. In (a) we show the appearance of spontaneous oscillations in the filament at 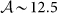 corresponding to rigid corkscrew rotation, followed by a transition at 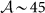 to flexible planar beating. In (b, inset) we show the increase in the unscaled amplitude with length.

Numerical simulations of Eq. (1) reveal two distinct oscillatory states (Figs. 1a, 1b, Supplementary Fig. S3a, Supplementary Videos 1 and 2). The first of these, seen in the range 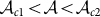, is a state in which the filament rotates rigidly in a corkscrew-like motion about the axis of the clamp. This rotational corkscrew motion is reminiscent of prokaryotic flagellar beating^4^,^5^. We show this motion in Fig. 1a over one time period of oscillation together with the projection of the filament on the plane perpendicular to the clamp axis. A section of the three-dimensional flow in a plane containing the clamp axis is shown in Figs. 2a and 2b. The net flow points in the direction opposite to the filament curvature and the entire flow pattern co-rotates with the filament. In the second state, seen for 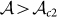, the filament beats periodically in a two-dimensional plane containing the axis of the clamp, with waves propagating from the clamp to the tip. This flexible beating is reminiscent of eukaryotic flagellar motion^2^,^3^,^7^,^9^,^10^. We show this motion in Fig. 1b over one time period of oscillation together with the projection of the filament on the plane perpendicular to the clamp axis. The projection is now a line, showing that motion is confined to a plane. A section of the three-dimensional flow in the plane of beating is shown in Figs. 3a and 3b. Two distinct types of filament conformations of opposite symmetry are now observed, corresponding to different parity of the conformation with respect to the perpendicular bisector of the line joining the two end points. In the *even* conformation (Fig. 3a), the flow points in the direction opposite to the curvature as in the corkscrew state. However, in the *odd* conformation (Fig. 3b), the flow has a centre of vorticity at the point of inflection of the filament. This centre of vorticity moves up the filament and is shed at the tip at the end of every half cycle. The critical activities scale as 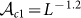 and 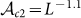, obtained from a Bayesian parameter estimation of data shown in Fig. S3b. The critical values depend only on the ratio *σ*_0_/*κ*, and not on *σ*_0_ and *κ* individually, as is clearly seen in Fig. 4a.

### Time period and amplitude scaling

The physical parameters determining the time period *T* of the oscillatory states are the active stresslet *σ*_0_, the bending modulus *κ*, the fluid viscosity *η* and the filament length *L*. Remarkably, variations of *T* in this four-dimensional parameter space collapse, when scaled by the active relaxation rate Γ*_σ_* = *σ*_0_/*ηL*^3^, to a one-dimensional scaling curve of the form 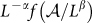. We show the data collapse at fixed *L* and varying relative activity *σ*_0_/*κ* in Fig. 4a while the scaling with system size is shown in Supplementary Fig. S2a. Our best estimates for the exponents, obtained from Bayesian regression, are *α* = 1.3 and *β* = −1.2 (Supplementary Fig. S2a). Qualitatively, at fixed relative activity the oscillation frequency decreases with increasing *L*, while at a constant *L* the oscillation frequency increases with increasing relative activity. This is in agreement with a simple dimensional estimate of the time period *T* ~ *ηL*^3^/*σ*_0_. For active beads with a stresslet of *σ*_0_ ~ 6 × 10^–18^ *Nm* in a filament of length *L* ~100 *μm*, our estimate of the time period gives a value of 170 *s*, which agrees in order of magnitude with experiment^11^. The amplitude of oscillation *ρ* obeys a similar scaling relation with *α* = –1.46, *β* = –1.2 (Fig. 4b, main panel). At fixed relative activity, *ρ* increases with increasing *L*, while at fixed *L*, it increases and then saturates at large relative activity. The amplitude in the planar beating state is marginally smaller than in the corkscrew rotating state (Fig. 4b, inset).

### Linear Stability and Hopf Bifurcation

**Figure 5:**
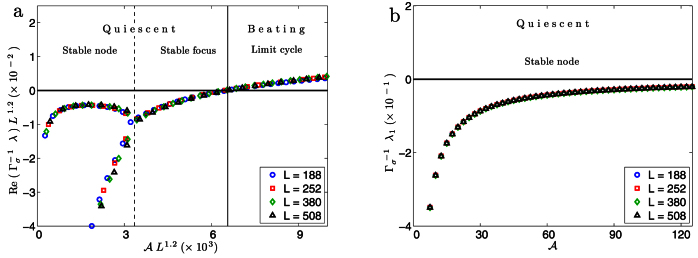
Variation of the largest scaled eigenvalues of the Jacobian matrix (a) including HI and (b) excluding HI. In (a) we see the transition from a stable node to a stable focus followed by a supercritical Hopf bifurcation from quiescence to a limit cycle. In (b) we see only a stable quiescent state. In the absence of HI the eigenvalue scaling is completely determined by 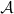. Comparing (a) and (b) it is clear that hydrodynamic instabilities due to HI are the main mechanism for spontaneous oscillations. (a) also shows that the rate of relaxation, given by the magnitude of the real part of the largest eigenvalue, decreases as we approach the bifurcation from below.

To better understand the nature of the hydrodynamic instability and the transition to spontaneous oscillation we performed a linear stability analysis^35^ of the straight filament. In absence of activity, 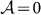, all eigenvalues of the Jacobian are real and negative and the filament has an overdamped relaxation to equilibrium. With increasing 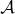, the two largest real eigenvalue pairs approach, converge, and become complex conjugate pairs. This corresponds to a transition from a stable node to a stable focus where the response changes from being overdamped to underdamped. The analysis reveals that the balance between hydrodynamic flow and linear elasticity has a non-monotonic variation. While the general trend is towards slower relaxations with increasing 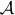 corresponding to the greater relative strength of the hydrodynamic flow, this is reversed in a small window of activity where the increasing activity produces faster relaxations. This can be clearly seen in Fig. 5a and Fig. S1a, where the rate of relaxation is given by the magnitude of the real part of the largest eigenvalue. With further increase of 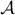 the complex eigenvalues approach the imaginary axis monotonically, crossing them at a critical value 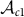 (Fig. 5a, Supplementary Figs. S1a and S1b, and Supplementary Video 3). Through this supercritical Hopf bifurcation, the stable focus flows into the limit cycle corresponding to the corkscrew rotation. The value of 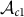 obtained from the linear stability analysis is in perfect agreement with that obtained from numerical simulation. As with the time-period and amplitude, the eigenvalues *λ* of the Jacobian obey scaling relations 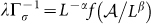 with *α* = 1.2 and *β* = –1.2.

### Importance of HI

To ascertain the importance of HI, we repeat the stability analysis on a local limit of our model. Here, the long-ranged contributions to the hydrodynamic flow from both elasticity and activity are neglected and only their short-ranged effects are retained (see Supplementary Text). We find that all eigenvalues remain real and negative for activity numbers corresponding to an order of magnitude greater than 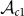, reflecting the stability of the quiescent state in the absence of HI (Fig. 5b).

## Discussions

Our work shows that simple chains of CABs, for instance of synthetic catalytic nanorods^31^,^32^,^33^,^34^, can show the spontaneous beating obtained previously in more complex systems like self-assembled motor-microtubule mixtures^11^ or externally actuated artificial cilia^36^,^37^,^38^,^39^. We emphasise that an experimental realisation of our system requires neither external actuation nor self-propulsion. The only chemomechanical requirement is that the CABs produce force-free and torque-free dipolar flows in the fluid. This makes them an attractive candidate for biomedical applications like targetted drug delivery. Our detailed prediction for the spatiotemporal dynamics of the hydrodynamic flows can be experimentally verified using particle imaging velocimetry^40^.

In summary, we have shown that a minimal filament model which includes elasticity, chemomechanical activity and HI, exhibits spontaneous emergent biomimetic behaviour reminiscent of the rhythmic oscillations of various prokaryotic and eukaryotic flagella^1^,^2^,^3^,^4^,^5^,^6^,^7^,^9^. Our results lead us to conclude that hydrodynamic instabilities due to internal active stresses are sufficient to induce spontaneous biomimetic beating in a clamped chemomechanically active filament.

## Methods

We calculate the RHS of Eq. (1) by a direct summation of the hydrodynamic Green's functions. Clamped boundary conditions are implemented at one end by fixing the position of the first particle and allowing the second particle to move only along the tangential direction. The equation of motion is integrated using a variable step method as implemented in ODE15s in Matlab. The hydrodynamic flows fields are obtained on a regularly spaced Eulerian grid by summing the individual contributions from each of the *N* particles. The linear stability analysis is performed by first numerically integrating the equations of motion to obtain the fixed point, then numerically evaluating the 3 *N* × 3 *N* Jacobian matrix at the fixed point, and finally computing the eigenvalues of the Jacobian matrix numerically. Simulations are carried out for different filament lengths *L* with bead numbers upto *N* = 128. The equilibrium bond length is taken to be *b*_0_ = 4. We choose *κ* in the range 0.0 to 1.0 and *σ*_0_ in the range 0.0 to 0.5. The initial condition is a random transverse perturbation applied to every particle. Random perturbations in the longitudinal direction relaxes at a much faster time-scale due to the stretching potential. The total integration time is typically 10Γ*_σ_*^–1^, where Γ*_σ_* = *σ*_0_/*ηL*^3^ is the active relaxation rate, and *η* is the viscosity, taken to be 1/6.

## Author Contributions

R.A. and P.B.S.K. designed research. A.L., R.S., S.G. and G.J. performed research. S.G., R.A., R.S. and P.B.S.K. wrote the manuscript.

## Supplementary Material

Supplementary Information Hydrodynamic instabilities provide a generic route to spontaneous biomimetic oscillations in chemomechanically active filaments : Supplementary Information

Supplementary Information Aplanar corkscrew-like rigid rotation of clamped active filament with flowfield

Supplementary Information Planar flexible periodic beating of clamped active filament with flowfield

Supplementary Information Hopf bifurcation in clamped active filament
