# Supplementary material for: Hydrodynamic instabilities provide a generic route to spontaneous biomimetic oscillations in chemomechanically active filaments
Source: Sci Rep. 2013 Jun 11;3:1964. doi: 10.1038/srep01964 (PMC3678140; doi:10.1038/srep01964)
Supplement: Supplementary Information — Hydrodynamic instabilities provide a generic route to spontaneous biomimetic oscillations in chemomechanically active filaments : Supplementary Information [file srep01964-s1.pdf]

Supplementary Information : Hydrodynamic instabilities provide a generic route to spontaneous biomimetic oscillations in chemomechanically active filaments

Abhrajit Laskar,[1] Rajeev Singh,[1] Somdeb Ghose,[1] Gayathri Jayaraman,[1] P. B. Sunil Kumar,[2] and R. Adhikari[1]

[1]*The Institute of Mathematical Sciences, CIT Campus, Chennai 600113, India*

[2]*Department of Physics, Indian Institute of Technology Madras, Chennai 600036, India*

(Dated: June 3, 2013)

MODEL

Following Ref. [1], we construct an active elastic filament by chaining active beads using potentials. We place N such beads at points $\mathbf{r}_1, \mathbf{r}_2, \ldots \mathbf{r}_N$ and define bond vectors $\mathbf{b}_m = \mathbf{r}_{m+1} - \mathbf{r}_m$ between adjoining beads. The potentials $U_\text{S}(\mathbf{b}_m) = \frac{1}{2}k(|\mathbf{b}_m| - b_0)^2$ and $U_\text{B}(\mathbf{b}_m, \mathbf{b}_{m+1}) = (\kappa/b_0)(1 - \cos\phi_m)$ model inextensibility and semiflexibility respectively, penalizing departures of the filament from the equilibrium bead-bead separation of b_0 or the bond-bond angle $\phi_m = 0$. Here k is the spring constant and κ is the bending modulus. Self-avoidance is enforced through a purely repulsive Lennard-Jones potential U_{LJ} which vanishes smoothly at a distance σ_{LJ}. The total potential $U(\mathbf{r}_1, \ldots, \mathbf{r}_N)$ is the sum of these three potentials. Stretching, bending and self-avoidance causes the total elastic force $\mathbf{f}_n = -\partial U(\mathbf{r}_1, \ldots, \mathbf{r}_N)/\partial \mathbf{r}_n$ to act on the n-th bead.

We model the activity using force-free and torque-free singularities [2–6], of which the second-rank symmetric stresslet tensor is the most dominant [7], and produces flows that decay inverse squarely with distance. With a tensorial strength $\boldsymbol{\sigma}_n$ and an axis of uniaxial symmetry $\mathbf{p}_n$, the stresslet can be parametrised as $\boldsymbol{\sigma}_n = \sigma_0(\mathbf{p}_n\mathbf{p}_n - \mathbb{I}/d)$ where d is the spatial dimension and σ_0 sets the scale of the activity. Extensile flows correspond to $\sigma_0 > 0$, while contractile flows correspond to $\sigma_0 < 0$. In the present case, we set $\mathbf{p}_n = \hat{\mathbf{t}}_n$, the local unit tangent vector, to reflect the tangential stresses generated by the active particles.

The filament exerts forces on the surrounding fluid due to its elasticity and activity. The resultant force density at the point $\mathbf{r}$ due to N active beads is given by summing over the Stokeslet and stresslet singularities,

$$\mathbf{F}(\mathbf{r}) = \sum_m \{\mathbf{f}_m \delta(\mathbf{r} - \mathbf{r}_m) + \nabla \cdot [\boldsymbol{\sigma}_m \delta(\mathbf{r} - \mathbf{r}_m)]\} \tag{1}$$

Integrating this on a surface enclosing the filament gives

$$\int d^3\mathbf{r}\, \mathbf{F}(\mathbf{r}) = \sum_m \left\{\mathbf{f}_m + \int d^3\mathbf{r}\, \nabla \cdot [\boldsymbol{\sigma}_m \delta(\mathbf{r} - \mathbf{r}_m)]\right\} = 0. \tag{2}$$

Since the elastic forces $\mathbf{f}_m$ are internal to the filament and obey Newton's third law, they cancel exactly on summation over all the beads. The active terms are total divergences and thus vanish on integration over a bounding surface. The symmetry of the stresslet tensor ensures that no angular momentum is added to the fluid. Consequently, the force density considered here does not add any *net* linear or angular momentum to the fluid. Models in which this fundamental constraint is not imposed will fail to correctly reproduce HI due to active energy transduction.

In the low Reynolds number regime, a force $\mathbf{f}(\mathbf{r}')$ and a stresslet $\boldsymbol{\sigma}(\mathbf{r}')$, placed at location $\mathbf{r}'$ in a three-dimensional unbounded fluid, produce flows $v^\text{el}_\alpha(\mathbf{r}) = O_{\alpha\beta}(\mathbf{r} - \mathbf{r}')f_\beta(\mathbf{r}')$ and $v^\text{ac}_\alpha(\mathbf{r}) = D_{\alpha\beta\gamma}(\mathbf{r} - \mathbf{r}')\sigma_{\beta\gamma}(\mathbf{r}')$ respectively.The Oseen and stresslet tensors are given by $O_{\alpha\beta}(\mathbf{r}) = (\delta_{\alpha\beta} + \hat{r}_\alpha\hat{r}_\beta)/8\pi\eta r$ and $D_{\alpha\beta\gamma}(\mathbf{r}) = (-\hat{r}_\alpha\delta_{\beta\gamma} + 3\hat{r}_\alpha\hat{r}_\beta\hat{r}_\gamma)/8\pi\eta r^2$ [8, 9] where $\hat{r}_\alpha = \mathbf{r}_\alpha/r$ and α, β are the Cartesian coordinates.

The velocity of the n-th bead is obtained by summing the force and activity contributions from all beads, including itself, to the fluid velocity at its location. Thus, we obtain the equation of motion [1]

$$\dot{\mathbf{r}}_n = \sum_{m=1}^{N} [\mathbf{O}(\mathbf{r}_n - \mathbf{r}_m) \cdot \mathbf{f}_m + \mathbf{D}(\mathbf{r}_n - \mathbf{r}_m) \cdot \boldsymbol{\sigma}_m]\,. \tag{3}$$

An isolated spherical bead with a force $\mathbf{f}$ acquires a velocity $\mu\,\mathbf{f}$ where μ is its mobility. By symmetry, an isolated spherical bead with a stresslet $\boldsymbol{\sigma}$ cannot acquire a velocity. Therefore, for $m = n$, $O_{\alpha\beta} = \mu\delta_{\alpha\beta}$ and $D_{\alpha\beta\gamma} = 0$. In the absence of activity, $\boldsymbol{\sigma}_n = 0$, and bending, $\kappa = 0$, the equation reduces to the Zimm model of hydrodynamic interactions of a polymer in a good solvent [10]. Dimensionally, the active and elastic forces are of the form σ_0/L and κ/L^2, where $L = (N-1)b_0$ is the length of the filament. The balance of these forces gives the dimensionless quantity $\mathcal{A} = L\sigma_0/\kappa$, which is also the ratio of the active and elastic rates of relaxation, respectively $\Gamma_\sigma = \sigma_0/\eta L^d$ and $\Gamma_\kappa = \kappa/\eta L^{d+1}$ [1]. The dynamics of the filament is completely captured by its length L and the relative active strength, given by this activity number $\mathcal{A}$.

In the free-draining approximation to our model, we ignore HI. Thus the velocity of the n-th bead due to elastic forces is $\mu\mathbf{f}_n$, where μ is the mobility. For the active velocity we retain contributions from immediate neighbours of the n-th bead. This gives a local equation of motion

$$\dot{\mathbf{r}}_n = \mu\mathbf{f}_n + \frac{\sigma_0}{4\pi\eta b_0^3}(\mathbf{b}_n - \mathbf{b}_{n-1}) \tag{4}$$

The parameter values used for the simulation and analysis are: fluid viscosity $\eta = 1/6$, radius of monomer $a =$

1, bond length $b_0 = 4a = 4$, spring constant $k = 1$ and the Lennard-Jones parameters $e = 0.001, r_{min} = b = 1$. Number of monomers varied from $N = 24$ to $N = 128$, while the remaining parameters κ is chosen in the range 0.0 to 1.0 and σ_0 in the range 0.0 to 0.5.

VIDEO TITLES AND CAPTIONS

Video S1 : *Aplanar corkscrew-like rigid rotation of clamped active filament with flowfield.*

Description : Filament motion for $L = 188$ and $\mathcal{A} = 25$, displaying rigid corkscrew-like rotation about the axis of the clamp over three time periods of oscillation. The time trace of the filament tip as well as a section of the three-dimensional flow in a plane containing the clamp axis are shown. The net flow points in the direction opposite to the filament curvature and the entire flow pattern co-rotates with the filament. This motion is reminiscent of prokaryotic flagellar beating.

Video S2 : *Planar flexible periodic beating of clamped active filament with flowfield.*

Description : Filament motion for $L = 188$ and $\mathcal{A} = 50$, displaying flexible periodic beating in a two-dimensional plane containing the axis of the clamp over two time periods of oscillation. A section of the three-dimensional flow in the plane of beating is shown. Two distinct types of filament conformations of opposite symmetry are observed as the filament oscillates. In the *even* conformation the flow points in the direction opposite to the curvature as in the corkscrew state. However, in the *odd* conformation the flow has a centre of vorticity at the point of inflection of the filament. This centre of vorticity moves up the filament and is shed at the tip at the end of every half cycle.

Video S3 : *Hopf bifurcation in clamped active filament.*

Description : Variation of real and imaginary parts of eigenvalues with activity number $\mathcal{A}$ for $L = 188$. The main panel shows the two largest eigenvalue pairs (red and blue pentagons) while the inset shows the entire spectrum. All eigenvalues are real and negative for $\mathcal{A} < 6$, beyond which the first pair converge and become complex conjugates, indicating the transition from the stable node to the stable focus. This pair crosses the imaginary axis at $\mathcal{A} \approx 12.5$, that is, at $\mathcal{A}_{c1}$, indicating the transition from the stable focus to a limit cycle through a Hopf bifurcation. The second pair replicates this entire behaviour at higher $\mathcal{A}$.

[1] G. Jayaraman, S. Ramachandran, S. Ghose, A. Laskar, M. Saad Bhamla, P. B. Sunil Kumar, and R. Adhikari, Phys. Rev. Lett. **109**, 158302 (2012).

[2] J. Blake, J. Fluid Mech **46**, 199 (1971).

[3] C. Brennen and H. Winet, Annu. Rev. Fluid Mech. **9**, 339 (1977).

[4] S. Ramaswamy, Annu. Rev. Condens. Mat. Phys. **1**, 323 (2010).

[5] M. Cates and F. MacKintosh, Soft Matter **7**, 3050 (2011).

[6] M. C. Marchetti, J.-F. Joanny, S. Ramaswamy, T. B. Liverpool, J. Prost, M. Rao, and R. Aditi Simha, ArXiv e-prints (2012), arXiv:1207.2929 [cond-mat.soft].

[7] A. T. Chwang and T. Y. Wu, J. Fluid Mech. **67**, 787 (1975).

[8] C. Pozrikidis, *Boundary Integral and Singularity Methods for Linearized Viscous Flow* (Cambridge University Press, Cambridge, 1992).

[9] S. Kim and S. Karrila, *Microhydrodynamics: Principles and Selected Applications*, Dover Civil and Mechanical Engineering Series (Dover Publications, 2005).

[10] M. Doi and S. F. Edwards, *The Theory of Polymer Dynamics* (Clarendon Press, Oxford, 1988); B. H. Zimm, J. Chem. Phys. **24**, 269 (1956).

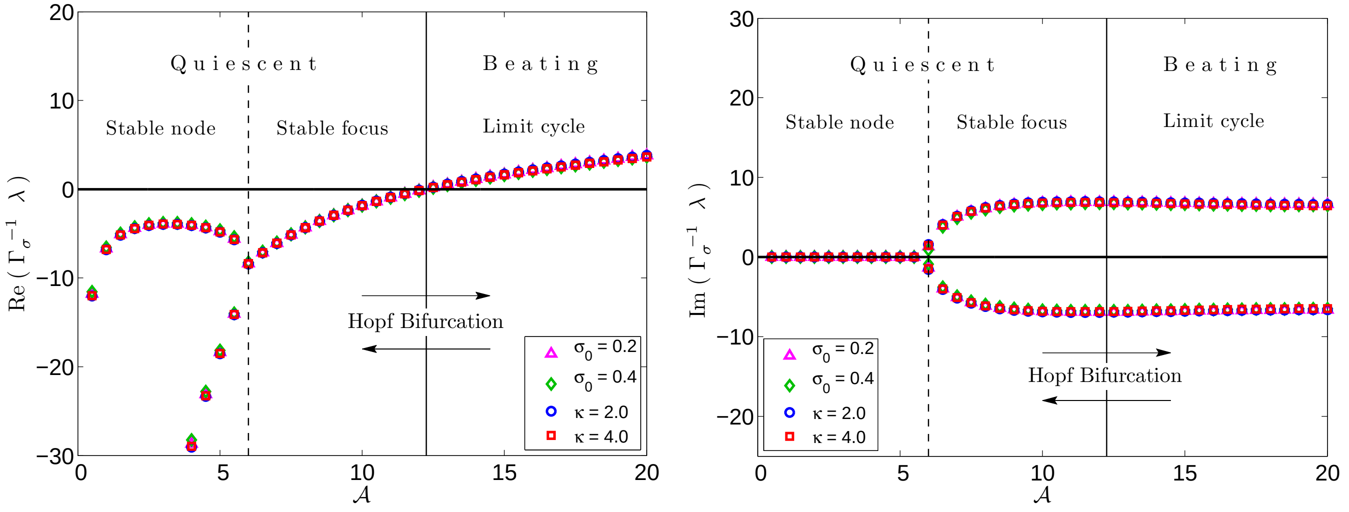

(a) (b)

FIG. S1. Variation of the scaled (a) real and (b) imaginary parts of the largest eigenvalues with $\mathcal{A}$, plotted for various values of κ and σ_0 with $L = 188$. Data is obtained from a linear stability analysis (LSA) of the filament model. The largest eigenvalue pairs converge at $\mathcal{A} \sim 6$ and become complex with negative real parts, signalling the transition from stable node to stable focus. Re(λ) become positive at $\mathcal{A}_{c1} \sim 12.5$ while Im(λ) varies smoothly with $\mathcal{A}$, indicating a Hopf bifurcation into a limit cycle.

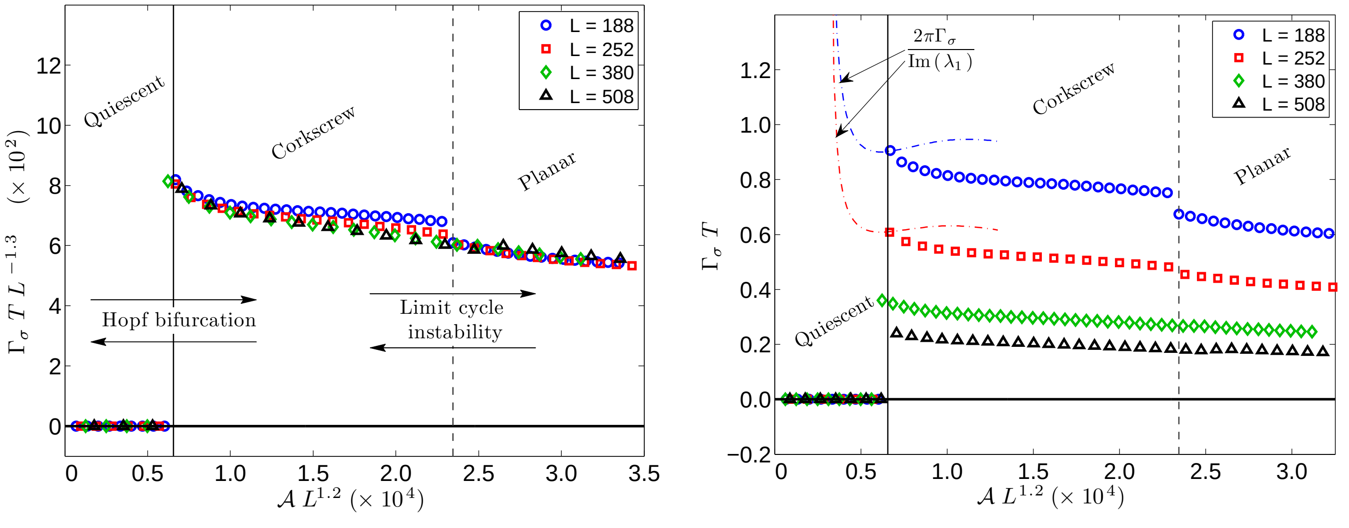

(a) (b)

FIG. S2. Variation of the nondimensionalised time period $\Gamma_\sigma T$ with activity number $\mathcal{A}$ (a) with and (b) without L scaling, obtained from numerical simulations. The dashed lines in (b) represents the variation of $2\pi\Gamma_\sigma/\mathrm{Im}(\lambda_1)$ with $\mathcal{A}$ for $L = 188$ and $L = 252$, data obtained from LSA. The rescaled plot of $\Gamma_\sigma T$ in (a) shows that its variations are well captured by a scaling form $L^{-\alpha} f(\mathcal{A}/L^{\beta})$, with $\alpha = 1.3$ and $\beta = -1.2$ estimated using Bayesian regression. The unscaled results in (b) shows that the time period is of the order of the active timescale Γ_σ^{-1}. The LSA estimate of the time period and the simulation result agree very well near the Hopf bifurcation point, and, predictably, deviates in the nonlinear regime.

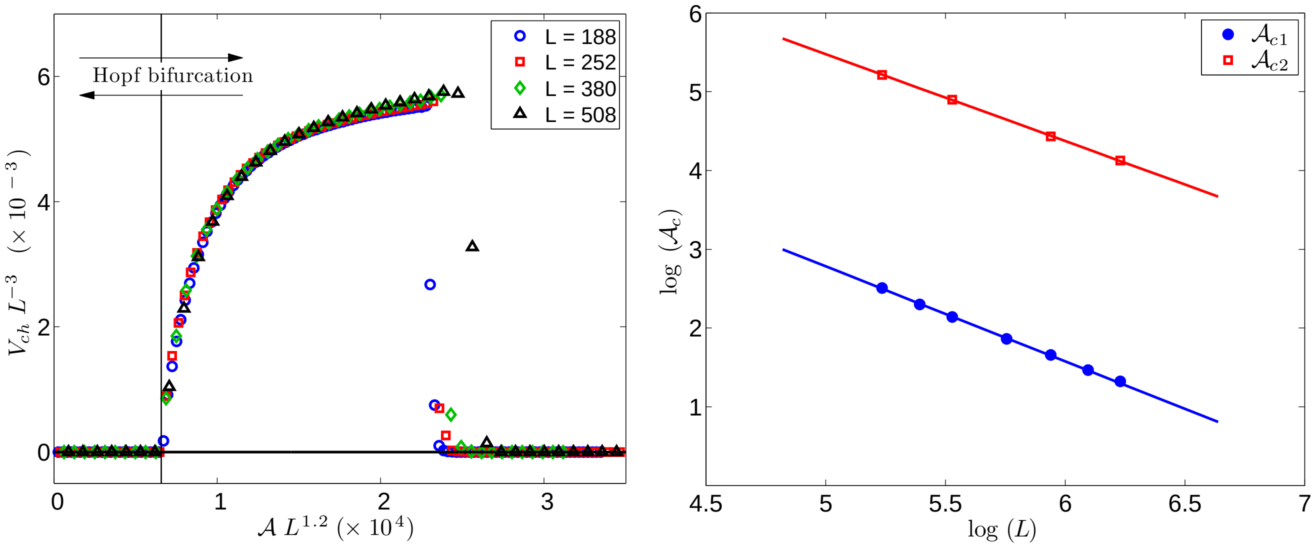

FIG. S3. (a) Variation of the rescaled volume of the convex hull $V_{\rm ch}$ of the filament with $\mathcal{A}$ showing the transition between aplanar rotations in the regime $\mathcal{A} < \mathcal{A}_{c2}$ and planar oscillations in the regime $\mathcal{A} > \mathcal{A}_{c2}$. (b) Variation of the transition points $\mathcal{A}_{c1}$ and $\mathcal{A}_{c2}$ with L exhibiting a scaling relation $\mathcal{A}_c \sim L^{\beta}$. Using Bayesian regression, we estimate $\beta = -1.2$ for $\mathcal{A}_{c1}$ and $\beta = -1.1$ for $\mathcal{A}_{c2}$, the different values responsible for the imperfect data collapse near the second transition. Symbols represent simulation data while solid lines represent the Bayesian estimate.
